# Comments on ‘The cardio–pancreatic axis in heart failure: from conceptual framework to empirical evidence’

**DOI:** 10.1093/eschf/xvag122

**Published:** 2026-05-04

**Authors:** Han Naung Tun, Omar Rahal, Ivan R Figueroa Baez, Omar Santana-Sánchez, Timothy Gardner

**Affiliations:** Geisel School of Medicine at Dartmouth, Hanover, NH 03755, USA; English Division, Medical University of Warsaw, Warsaw, Poland; San Juan City Hospital Internal Medicine Training Program, San Juan 00936, Puerto Rico; VA Caribbean Healthcare System, Internal Medicine Training Program, San Juan 00921, Puerto Rico; Center for Digestive Health, Dartmouth-Hitchcock Medical Center, Lebanon, NH 03756, USA

**Keywords:** HFpEF, Cardio-Pancreatic axis, Heart-Liver-Pancreatic axis, Heart failure

To the Editor,

We would like to thank the authors Dams *et al*. for providing reinforcing evidence, which shifts our conceptual framework of the Heart-Liver-Pancreatic axis into a clinically tangible entity. The editorial mentions the presence of the Heart-Liver-Pancreatic axis across the heart failure spectrum. This is valid as a congestive heart, as shown in our review, leads to congestive hepatopathy and a resulting pancreatic congestion, and in both organs, acute heart failure decompensation leads to acute liver injury and acute pancreatic ischaemia. This is why increased central venous pressure and splanchnic venous congestion plays a central part and it is an essential link between the liver and pancreas. A mini review discusses six trials in which a total of 259 HFpEF patients have undergone greater splanchnic nerve denervation, and it was found to be beneficial.^1^ The pancreas may represent a critical yet under recognized organ within the systemic pathophysiology of HFpEF, with potential implications for metabolic regulation, nutrition, and clinical outcomes.

The pancreas is central in the Heart-Liver-Pancreatic axis, and in our review, we primarily focused on acute and chronic pancreatitis. We would like to add that the pancreas is a highly immunogenic, and a clear example that delineate this is seen when discussing pancreatic and kidney transplant in CKD patients due to type 1 diabetes, where pancreatic biopsies have higher expression of MHC-II in comparison to kidney biopsies.^2^ Inflammation of the pancreas seen in acute pancreatitis, as well as pancreatic ductal carcinoma triggers a systemic immune response, and it can be assessed by blood cell count ratios.^3^ A study on 37 patients with acute pancreatitis, more than 2/3 of these patients had elevated NT-pro-BNP which demonstrate direct cardiac involvement.^4^ This immunogenicity may explain the strong systemic response of the pancreas and the closely association of HFpEF and systemic inflammation.

This raises important questions, including whether patients with HFpEF should be screened for pancreatic exocrine dysfunction using faecal elastase. We agree that its accuracy is often limited in mild insufficiency and may be further confounded by the altered bowel habits commonly observed in heart failure.^5^ These factors may complicate the interpretation of FE-1 and limit its reliability in this population.^5^ However, data regarding the interpretation of FE-1 in patients with heart failure remain limited. Future studies should therefore incorporate additional functional assessments of pancreatic exocrine function and explore pragmatic trials of pancreatic enzyme replacement therapy in patients with a potential cardio-pancreatic cachectic phenotype.

Our generated hypothesis and proposed axis warrant a novel phenotypic classification of HFpEF, especially when historically used medication in heart failure were not very effective, and novel medications such as SGLT2 inhibitors and GLP-1 receptor agonists have considered a foundational for the management of HFpEF. The cardio–hepato–pancreatic axis provides a biologically plausible framework that helps explain the heterogeneity, comorbidity burden, and therapeutic challenges of HFpEF, highlighting the pancreas as a potentially important but under recognized target organ. Accordingly, future HFpEF research protocols should incorporate pancreatic evaluation together with portal haemodynamic validation to better define this axis and its clinical implications.

## Declarations

### Data Availability

No data were generated or analysed for this manuscript.
